# Supplementary material for: Prostaglandin E2 inhibits adipogenesis through the cilia-dependent activation of ROCK2
Source: J Cell Sci. 2025 Oct 24;138(20):jcs264193. doi: 10.1242/jcs.264193 (PMC12582440; doi:10.1242/jcs.264193)
Supplement: Supplementary information [file joces-138-264193-s1.pdf]

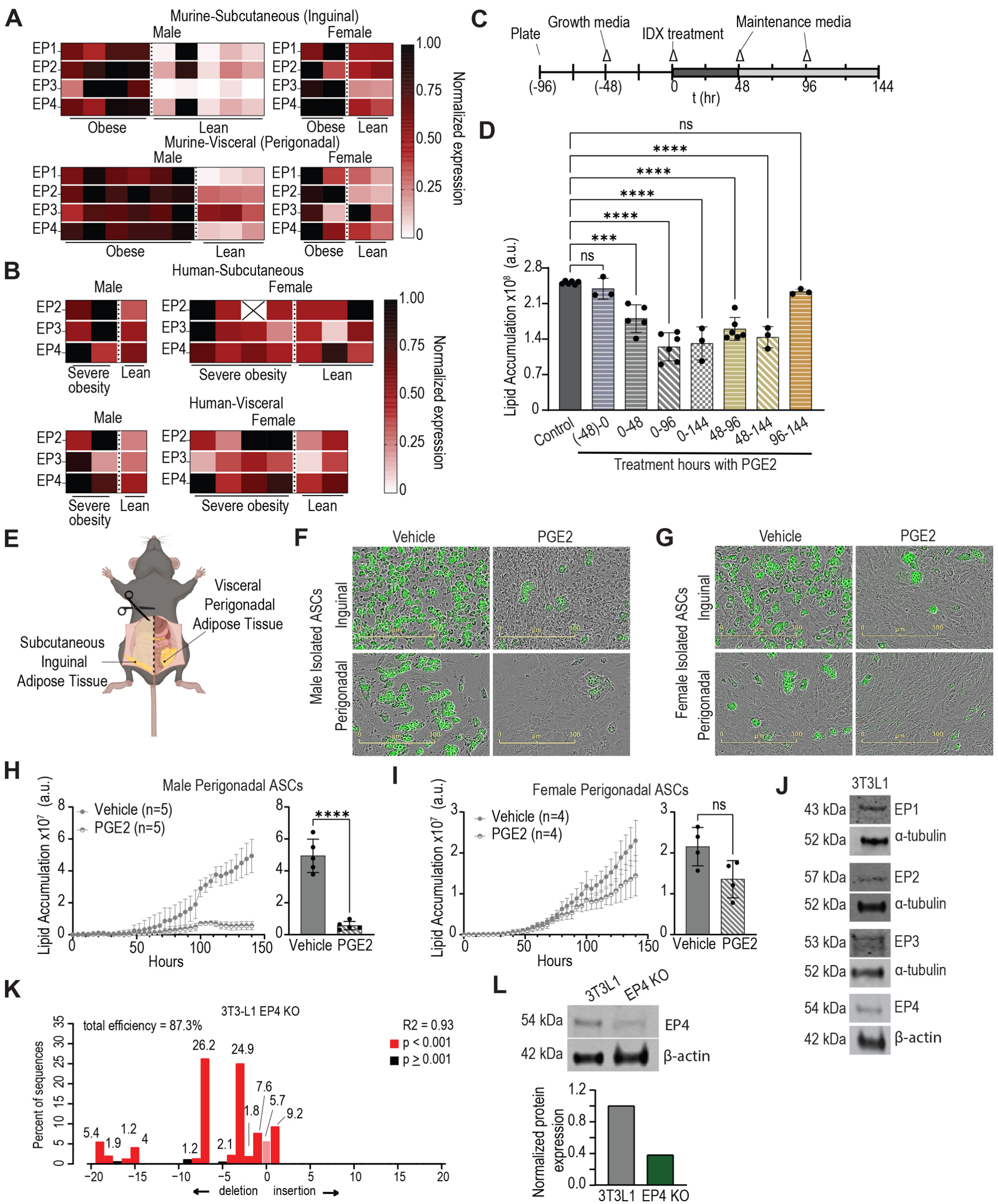


Journal of Cell Science • Supplementary information

**Fig. S1.** (A, B) Analysis of single-nucleus RNA sequencing data from(Emont *et al.*, 2022) demonstrating the elevated expression of PGE2 receptors EP1-4 in ASCs from obese versus lean white adipose tissue of mice (A) and human samples (B). Receptor expression has been normalized for each group. (C) Schematic of treatment with the IDX cocktail. Δ represent media changes during differentiation with the media indicated above. (D) Time-dependent treatments with 20µM PGE2 show inhibitory activity varies depending on window of treatment. Indicated time windows in hours and relative to t=0h being the initiation of adipogenesis as illustrated in (C). (E) Schematic depicting the subcutaneous inguinal and visceral perigonadal adipose tissue depots. Created in BioRender by Lee, M., 2025. https://BioRender.com/zb10prr. This figure was sublicensed under CC-BY 4.0 terms. (F, G) Representative images of lipid droplets in ASCs isolated from inguinal and perigonadal depots of male (F) and female (G) mice and differentiated in the presence of 20µM PGE2 or vehicle control during the first 72 hours of adipogenesis. (H, I) Isolated perigonadal ASCs from male (H) or female (I) mice are sensitive to 20µM PGE2 added during the first 72 hours of IDX-stimulated adipogenesis. n=number of independent ASC isolations, each pooling of 2-4 mice. (J) Immunoblot showing expression of EP receptors in 3T3-L1 cells. (K) TIDE sequence analysis assessing knockout efficiency in EP4 knockout cells. $R^2$ measures proportion of the population accounted for in sequencing window, and total efficiency measures the significant cell populations with frame-shift mutations within the accounted for population. (L) Immunoblot showing EP4 expressing in 3T3-L1 cells and EP4 knockout cells, with quantification showing normalized expression. (D, H-I) All data are mean + SD, each data point shows an independent experiment. p-values calculated using one-way ANOVA followed by Tukey’s multiple comparison test (D) or student’s t-test (H,I) (ns=not significant, p<*0.05, **0.01, ***0.001, ****0.0001).

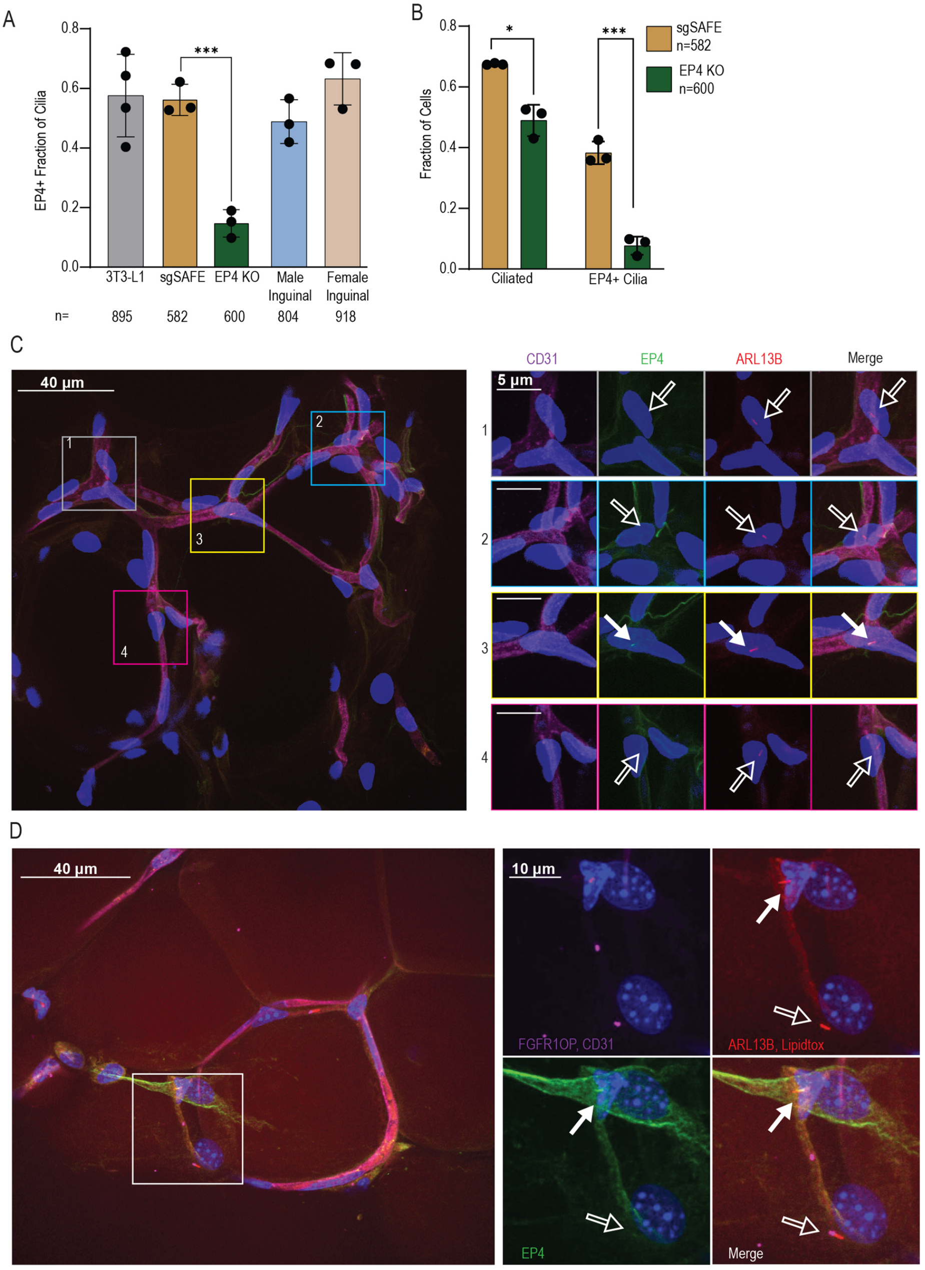


Journal of Cell Science • Supplementary information

**Fig. S2.** (A) Fraction of cilia from 3T3-L1 control cells, sgSAFE cells, 3T3-L1 EP4 knockout cells, and primary ASC cells that are positive for EP4. 3T3-L1 and primary cell data from Figure 2A-C, highlighting consistent ciliary localization across ASCs despite some differences in total ciliation. (B) EP4 antibody validation in undifferentiated 3T3-L1 EP4 knockout and sgSAFE cells. Note that the EP4 knockout cell line is a heterogenous, non-clonal population since 3T3-L1 cells lose their adipogenic capacity when single cell sorted, which is required to generate clonal cell lines. The vast majority of EP4 is depleted in the knockout cell line. Fraction of total cells that are ciliated or have ciliary EP4. Fraction of cilia that are EP4-positive is shown in (A). (C) 3D surface rendering of whole mount perigonadal adipose tissue stained for the endothelial cell marker CD31, EP4, cilia marker ARL13B, and DAPI. Colored boxes and numbers coordinate with insets highlighting primary cilia. Ciliary EP4 denoted by closed arrows, primary cilia without EP4 marked by open arrow. (D) Maximum intensity Z-stack projection of perigonadal adipose tissue sections stained with CD31, FGFROP, ARL13B, the lipophilic dye HSC LipidTOX (red), EP4, and DAPI. Mature adipocytes are lipid-filled and are enriched for HSC LipidTOX. Colocalization of EP4 with primary cilia is denoted by closed arrows, open arrows mark cilia without EP4. Tissue samples for (C) and (D) are from separate mice. (A-B) All data are mean ± SD and each data point represents an independent experiment. Each independent primary ASC isolation pooled 2-4 mice. n=total cells counted per condition. P-values calculated using student's t-test (ns=not significant, p<*0.05, **0.01, ***0.001, ****0.0001).

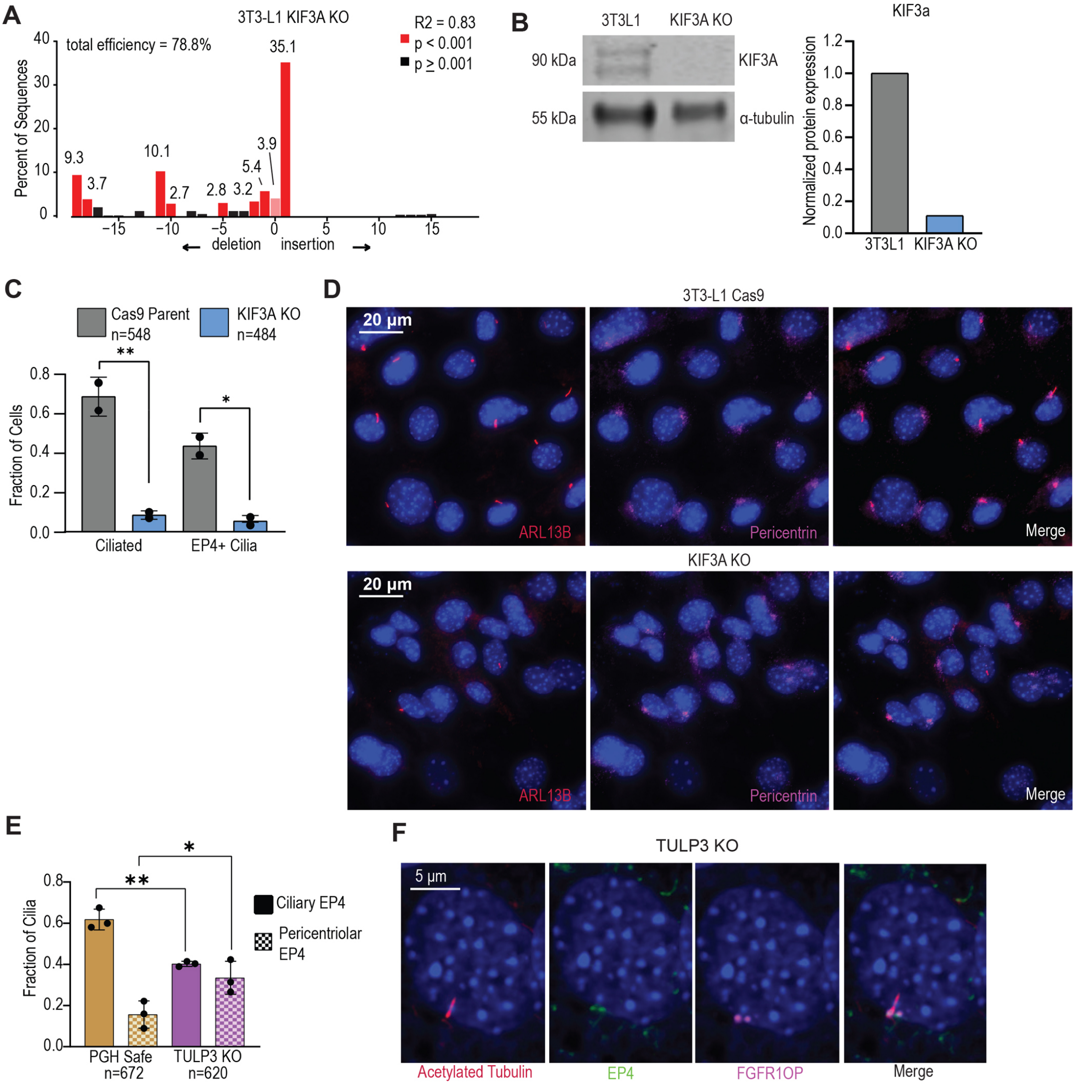


Journal of Cell Science • Supplementary information

**Fig. S3.** (A) TIDE sequence analysis assessing knockout efficiency in KIF3a knockout cells. $R^2$ measures proportion of the population accounted for in sequencing window, and total efficiency measures the significant cell populations with frame-shift mutations within the accounted for population. (B) Immunoblot of KIF3A protein in 3T3-L1 cells and KIF3A knockout cells, with quantification showing normalized expression. (C) Fraction of cells with cilia and EP4-positive cilia in Cas9-expressing 3T3-L1 cells and KIF3A knockout cells. KIF3A knockout cells are largely without cilia, but those that retain cilia retain the localization of EP4. (D) Representative immunofluorescence images of KIF3A knockout cells lacking primary cilia, marked with antibodies for the cilia marker ARL13B (red) and centrosome marker pericentrin (magenta). All cells have a nucleus and a centrosome (pericentrin), but the fraction of cells that have a centrosome and a primary cilium is reduced in the KIF3A knockouts. (E) TULP3 knockout cells have reduced EP4 trafficking into cilia, but accumulate EP4 at the base of the cilium adjacent to the centrosome. (F) Representative immunofluorescence images of TULP3 knockout cells with pericentriolar EP4 accumulation. Images were stained with antibodies for the cilia marker acetylated tubulin (red), EP4 (green), and FGFR1OP (magenta). Acetylated tubulin was used to mark the primary cilium, since ARL13B trafficking to cilia is reduced in TULP3 KOs. (C, E) All data are mean ± SD and each data point represents an independent experiment. n=total cells counted for each condition. P-values calculated using Student's T-test (C) or one-way ANOVA followed by Tukey's multiple comparison test (E) (ns=not significant, p<*0.05, **0.01, ***0.001, ****0.0001).

Journal of Cell Science • Supplementary information

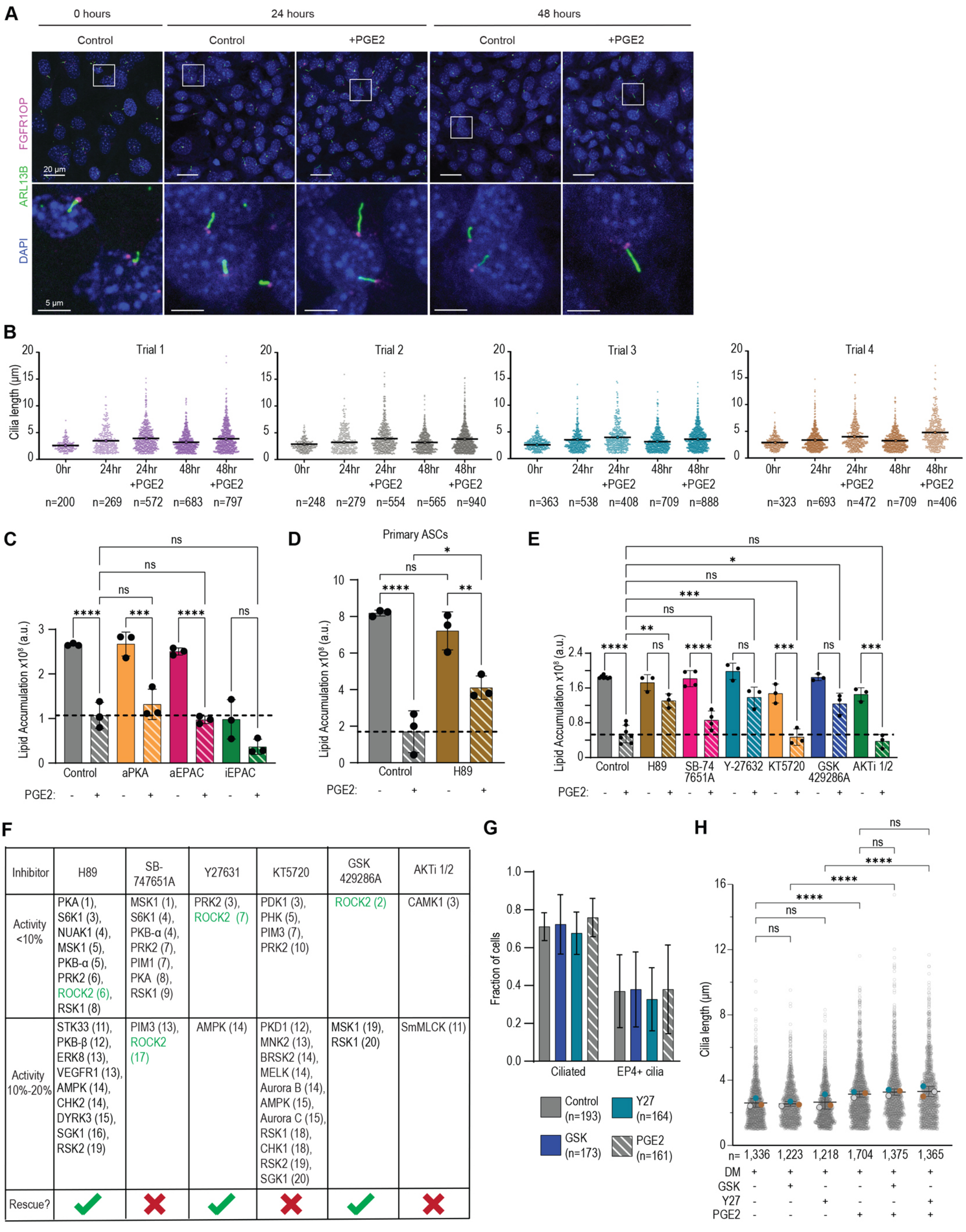
A
0 hours
24 hours
48 hours
Control
Control
+PGE2
Control
+PGE2
FGFR1OP
ARL13B
DAPI
20 µm
5 µm
B
Trial 1
Trial 2
Trial 3
Trial 4
Cilia length (µm)
0hr 24hr 24hr +PGE2 48hr 48hr +PGE2
n=200 n=269 n=572 n=683 n=797
n=248 n=279 n=554 n=565 n=940
n=363 n=538 n=408 n=709 n=888
n=323 n=693 n=472 n=709 n=406
C
Lipid Accumulation x10⁸ (a.u.)
Control aPKA aEPAC iEPAC
PGE2: - + - + - + - +
ns
****
***
D
Primary ASCs
Control H89
PGE2: - + - +
E
Control H89 SB-74 7651A Y-27632 KT5720 GSK 429286A AKTi 1/2
PGE2: - + - + - + - + - + - + - +
F
Inhibitor | H89 | SB-747651A | Y27631 | KT5720 | GSK 429286A | AKTi 1/2
Activity <10% | PKA (1), S6K1 (3), NUAK1 (4), MSK1 (5), PKB-α (5), PRK2 (6), ROCK2 (6), RSK1 (8) | MSK1 (1), S6K1 (4), PKB-α (4), PRK2 (7), PIM1 (7), PKA (8), RSK1 (9) | PRK2 (3), ROCK2 (7) | PDK1 (3), PHK (5), PIM3 (7), PRK2 (10) | ROCK2 (2) | CAMK1 (3)
Activity 10%-20% | STK33 (11), PKB-β (12), ERK8 (13), VEGFR1 (13), AMPK (14), CHK2 (14), DYRK3 (15), SGK1 (16), RSK2 (19) | PIM3 (13), ROCK2 (17) | AMPK (14) | PKD1 (12), MNK2 (13), BRSK2 (14), MELK (14), Aurora B (14), AMPK (15), Aurora C (15), RSK1 (18), CHK1 (18), RSK2 (19), SGK1 (20) | MSK1 (19), RSK1 (20) | SmMLCK (11)
Rescue?
G
Fraction of cells
Ciliated
EP4+ cilia
Control (n=193)
Y27 (n=164)
GSK (n=173)
PGE2 (n=161)
H
Cilia length (µm)
n= 1,336 1,223 1,218 1,704 1,375 1,365
DM
GSK
Y27
PGE2

**Fig. S4.** (A) Representative images depicting changes in 3T3-L1 primary cilia length during adipogenesis with or without PGE2 treatments. Stained with DAPI (blue), ARL13B (green), and FGFR1OP (magenta). Second row are insets. (B) Individual cilia length measurements from four independent trials illustrates congruity between repeats. Each open circle represents the length of an individual primary cilium, with horizontal bars representing the average for each condition. Colors for each trial are the same as in Figure 4A. (C) 3T3-L1 cells differentiated in the presence or absence of 20μM PGE2 and different pharmacological agents during the first 48 hours of adipogenesis demonstrate effector proteins downstream of cAMP induction do not mediate PGE2's effect on adipogenesis. Treatment with the PKA activator N6-Benzyl cAMP (100μM) or EPAC activator 8-pCPT-O-Methyl (50μM) on their own do not recapitulate the effect of PGE2, nor do they rescue adipogenesis during cotreatment. The EPAC inhibitor ESI-09 (20μM) demonstrates the necessity of EPAC during adipogenesis. Dotted line marks average endpoint lipid content in vehicle-treated control cells treated with PGE2. (D) ASCs isolated from inguinal white adipose tissue differentiated in the presence or absence of 20μM PGE2 and 10μM H89. H89 partially rescues adipogenesis from PGE2 treatment. Dotted line marks average endpoint (192 hours) lipid content in vehicle-treated control cells treated with PGE2. (E) Kinase inhibitors show variable ability to rescue PGE2 treatment during adipogenesis. 3T3-L1 cells cotreated with 20μM PGE2 and the indicated inhibitor during the first 48 hours of adipogenesis reveal that H89 (10μM), Y-27632 (20μM), and GSK429286A (1μM) rescue adipogenesis in the presence of PGE2. SB747651A (3.13μM), KT5720 (0.5μM), and AKTi 1/2 (0.13μM) do not rescue PGE2. Inhibitor concentrations were selected to have minimal effect on adipogenesis in the absence of PGE2. (F) Table of inhibitors from (E) indicates the percent activity remaining (parentheses) for all kinase targets shared between the top 17 targets of H89 and the listed kinase inhibitor. Green text indicates the degree to which ROCK2 is inhibited by each kinase. Checkmarks denote a rescue of adipogenesis with PGE2 treatment while an X denotes no significant change as shown in (E). (G) Fraction of 3T3-L1 cells with cilia and EP4-positive cilia following 48 hours of adipogenesis in the presence of ROCK inhibitors Y-27632(20μM) and GSK429286A (1μM) with and without PGE2 (20μM) cotreatment. Data presented as mean $\pm$ SD from images chosen at random from three independent trials. n=total cells counted per condition.(H) Cilia length quantification of 3T3-L1 cells from treatment conditions in (G). Each open circle represents an individual cilium length from one of three independent trials, with trial averages included as solid-color circles. n=total cilia counted for each condition.  Mean $\pm$ SD of repeat averages included as solid lines on top of each condition. (C-E) All data are mean $\pm$ SD and each data point represents an independent experiment. Each independent primary ASC isolation pooled 2-4 mice. All p-values calculated using one-way ANOVA followed by Tukey's multiple comparison test (ns=not significant, p<*0.05, **0.01, ***0.001, ****0.0001).

Journal of Cell Science • Supplementary information

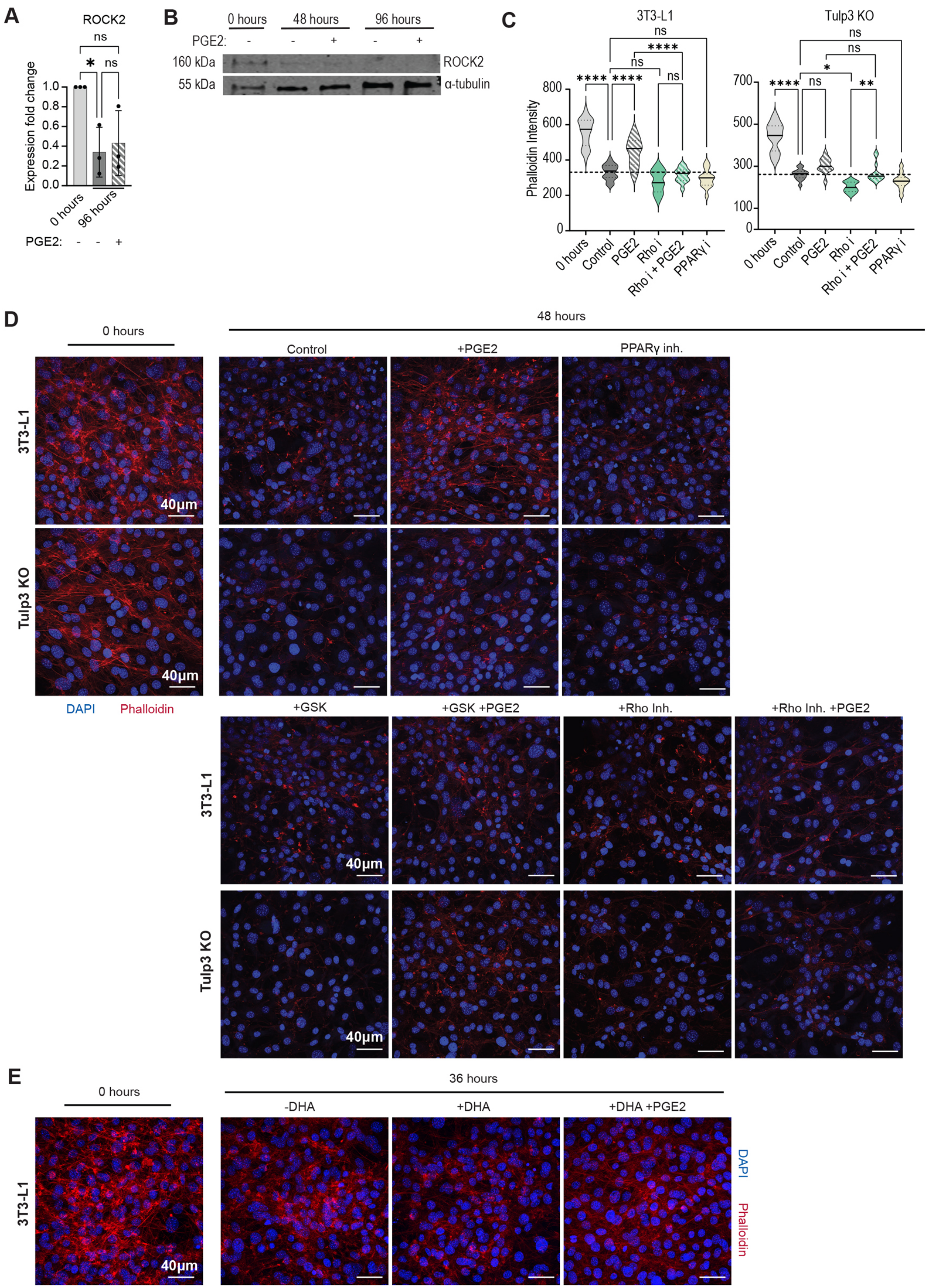


Journal of Cell Science • Supplementary information

**Fig. S5.** (A) Relative change in mRNA expression of ROCK2 during adipogenesis. PGE2 treatment does not alter this downregulation. (B) Immunoblot depicting change in ROCK2 protein levels during adipogenesis with or without 20µM PGE2 treatments. (C) Violin plots quantifying actin intensity from phalloidin staining in 3T3-L1 (*left*) and TULP3 knockout cells (*right*) following 48 hours of adipogenesis with PGE2 (20µM), Rho Inhibitor (0.5µg/ml), the PPARγ inhibitor T0070907 (1µM), or various cotreatments listed. Violin plots represent all individual images from 3-4 independent repeats (6 images per trial), with median indicated by the solid line and quartiles indicated by the dotted lines. Dashed lines represent the average for the control treatment at 48 hours. (D) Representative images from (C) and Figure 5C-E. (E) Representative images of 3T3-L1 cells differentiated with an attenuated differentiation cocktail supplemented with a ciliary GPCR agonist to induce adipogenesis with and without 20µM PGE2 cotreatment. Overall adipogenesis is decreased with this attenuated cocktail (Figure 3D) though actin stress fibers are largely maintained with PGE2 treatment. (A) Data are mean ± SD, each data point represents an independent experiment. (A, C) All P-values calculated using one-way ANOVA followed by Tukey's multiple comparison test (ns=not significant, p<*0.05, **0.01, ***0.001, ****0.0001).

## Figure S1J

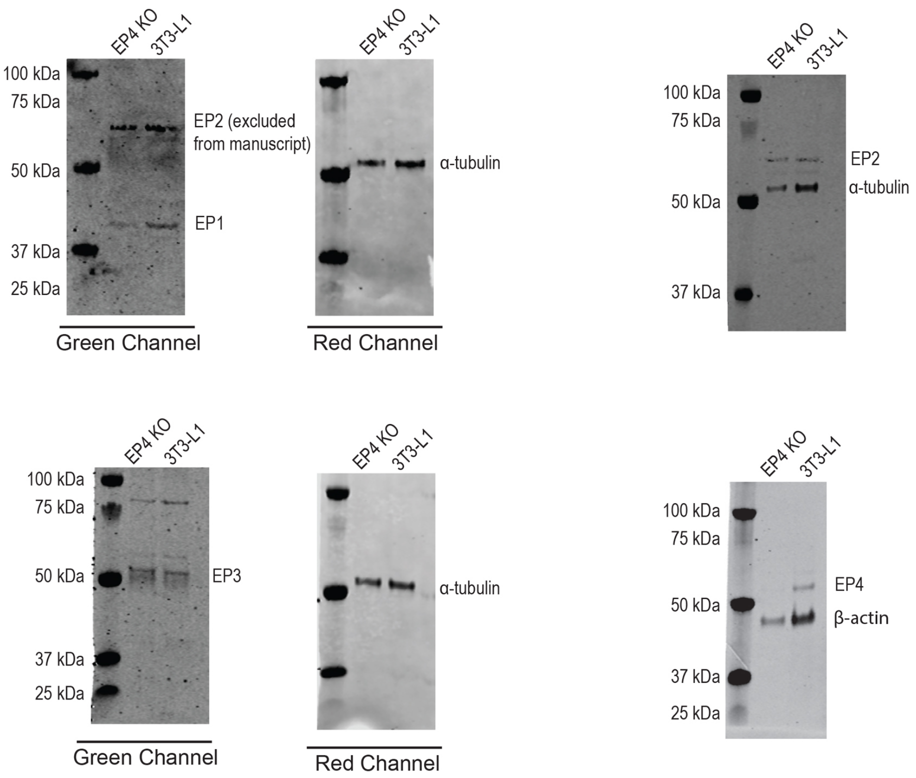


## Figure S1L

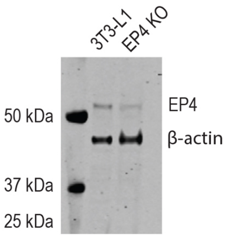


## Figure S3B

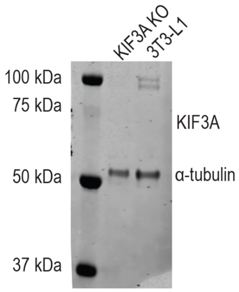


## Figure S5B

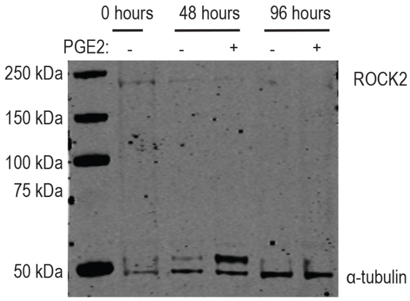

**Fig. S6.** Uncropped immunoblots from figures S1J, S1L, S3B, and S5B. Approximate ladder sizes are provided for each blot, and blotted proteins are indicated adjacent to their band. In cases of blots overlapping, images from separate trials have been provided for clarity.

**Table S1.** Small molecule reagents used to interrogate adipogenesis. Each reagent is listed with its target, role, source, treatment window, and concentration used in the manuscript.

| Compound | Primary target(s) | Role | Catalog number | Treatment window | Final concentration used |
|---|---|---|---|---|---|
| SC-19220 | EP1 | Antagonist | Cayman Chemical, 14060 | (-24hr)-48hr | 10 µM |
| PF-04418948 | EP2 | Antagonist | Cayman Chemical, 15016 | (-24hr)-48hr | 10 µM |
| L-798,106 | EP3 | Antagonist | Cayman Chemical, 11129 | (-24hr)-48hr | 5 µM |
| MF498 | EP4 | Antagonist | Cayman Chemical, 15973 | (-24hr)-48hr | 25 µM |
| MF766 | EP4 | Antagonist | Cayman Chemical, 36076 | (-24hr)-48hr | 50 µM |
| CAY10598 | EP4 | Agonist | Cayman Chemical, 13281 | 0-48hr | 50 µM |
| Docosahexaenoic acid | FFAR4 | Agonist | Millipore Sigma, D2534 | 0-48hr | 100 µM |
| Rp-cAMPs | PKA | Competative Inhibitor and antagonist | Cayman Chemical, 16985 | 0-96 hr | 25 µM |
| Rp-8-Br-cAMPs | PKA | Competative Inhibitor and antagonist | Cayman Chemical, 21584 | 0-96 hr | 25 µM |
| H89 | PKA | Inhibitor | Cayman Chemical, 10010556 | 0-96 hr, 0-48 hr, 0-72 hour (primary cells) | 10 µM |
| 8-pCPT-O-Methyl | EPAC | Selective activator | Cayman Chemical, 17143 | 0-48 hr | 50 µM |
| ESI-09 | EPAC | Selective inhibitor | Cayman Chemical, 19130 | 0-48 hr | 20 µM |
| N6-Benzyl cAMP | PKA | Selective activator | Cayman Chemical, 18819 | 0-48 hr | 100 µM |
| GSK429486A | ROCK1/ ROCK2 | Inhibitor | MedChemExpress, HY-11000 | 0-48 hr | 1 µM |
| SB74651A | MSK1 | Inhibitor | MedChemExpress, HY-110313 | 0-48 hr | 3.13 µM |
| Y-27632 | ROCK1/ ROCK2 | Inhibitor | Cayman Chemical, 10005583 | 0-48 hr | 20 µM |
| KT5720 | PKA | Inhibitor | Cayman Chemical, 10011011 | 0-48 hr | 0.5 µM |
| AKTi 1/2 | AKT1/ AKT2 | Inhibitor | Cayman Chemical, 14870 | 0-48 hr | 0.13 µM |
| Rho Inhibitor I | Rho GTPases | Inhibitor | Cytoskeleton, Inc., CT04 | 0-48 hr | 0.5 µg/ml |
| Rho Activator II | Rho GTPases | Activator | Cytoskeleton, Inc., CN03 | 0-48 hr | 0.25 µg/ml |

**Table S2.** Oligos used for cloning. List of all short oligo sequences used for generating and sequencing knockout cell lines in the manuscript.

Oligos used for generating sgRNA expressing pCMB plasmids

| Gene | Oligo Name F | Oligo Name R | ID | Oligos | |
|---|---|---|---|---|---|
| EP4/ Ptger4 | sgMDL001_EP4_Mm_F1 | sgMDL002_EP4_Mm_R1 | DrugTargetsKinasePhosphatase_Ptger4_ENSMUSG00000039942_134605.1 | TTGGTTCATCTTCGGGGTGGTGTTTAAGAGC | TTAGCTCTTAAACACCACCCCGAAGATGAACCAACAAG |
| Kif3a | sgMDL009_Kif3a_Mm_F2 | sgMDL010_Kif3a_Mm_R2 | StressProteostasis_Kif3a_ENSMUSG00000018395_97354.2 | TTGGGCGTTCGAGCAGTACCGGTTTAAGAGC | TTAGCTCTTAAACCGGTACTGCTCGAACGCCCAACAAG |

Primers used to amplify sgRNA targeted genomic regions and sanger sequencing primers

| Primer name | Seq (5->3) | Product size | Purpose |
|---|---|---|---|
| prUMDL001 | GGTGCTCTTGACAGAACGGA | ~1200 | Primers amplifying exon 2 of Ptger4/EP4 (sgRNA target) |
| prUMDL002 | AGGAATCAGGGAAAAGGAGGG | | |
| prUMDL011 | AGTTGGATCTGGGTGCTCTC | ~1100 | Nexted sequencing primer for exon 2 of Ptger/EP4 |
| | | | |
| prUMDL005 | ggctgccctagaatgttcca | ~700 | Primers amplifying exon 3 of Kif3a (sgRNA target) |
| prUMDL006 | AACGGGACTGCACTGACATA | | |
| prUMDL007 | ccattgtgcccagGTGTAGA | ~600 | Nested sequencing primer for exon 3 from Kif3a |

Journal of Cell Science • Supplementary information
